# Genome Sequence of *Klebsiella pneumoniae* CICC10011, a Promising Strain for High 2,3-Butanediol Production

**DOI:** 10.1128/genomeA.00802-15

**Published:** 2015-07-23

**Authors:** Ying-Jia Tong, Xiao-Jun Ji, Lu-Gang Liu, Meng-Qiu Shen, He Huang

**Affiliations:** State Key Laboratory of Materials-Oriented Chemical Engineering, College of Biotechnology and Pharmaceutical Engineering, Nanjing Tech University, Nanjing, People’s Republic of China

## Abstract

*Klebsiella pneumoniae* CICC10011, a promising 2,3-butanediol producer, has received much attention because of its high productivity. Here, the first draft genome sequence of this efficient strain may provide the genetic basis for further insights into the metabolic and regulatory mechanisms underlying the production of 2,3-butanediol at a high titer.

## GENOME ANNOUNCEMENT

Due to its extensive industrial applications in various fields, such as foods, cosmetics, pharmaceuticals, transport fuels, and agrochemical industries (1–3), 2,3-butanediol (2,3-BD) is a promising biobased bulk chemical. Many microorganisms, such as *Klebsiella pneumoniae*, *Klebsiella oxytoca*, *Enterobacter cloacae*, and *Serratia marcescens* (1, 2, 4–6), are known to be able to produce 2,3-BD. Among all these strains, *K. pneumoniae* was the first to be identified (1, 7) and is one of the best organisms that has shown the potential for industrial 2,3-BD production because of its more complete fermentation, broad substrate spectrum (hexoses, pentose, certain disaccharides, and uronic acid derived from the hydrolysates of hemicellulosic and cellulosic materials), cultural adaptability, and high efficiency (1, 7, 8). As a commercial strain, *K. pneumoniae* CICC10011 is regarded as a highly efficient producer of 2,3-BD (9, 10). Given the limited genetic information available on the metabolic mechanism underlying the formation of 2,3-BD, research about this strain is mainly focused on fermentation conditions and fermentation substrates rather than metabolic engineering. We therefore sequenced and analyzed the genome of strain *K. pneumoniae* CICC10011 to provide the genetic basis for the production of 2,3-BD at a high titer.

Here, we present the draft genome sequence of strain *K. pneumoniae* CICC10011, obtained using the Illumina HiSeq 2500 system at the Chinese National Human Genome Center, Shanghai, China. The reads were trimmed and *de novo* assembled with Velvet version 1.2.03 (11). Open reading frames (ORFs) were identified using the program Glimmer 3.02 (http://ccb.jhu.edu/software/glimmer/index.shtml). These ORFs were further annotated by comparison with the NCBI nr, KEGG, and Clusters of Orthologous Groups (COG) databases. The draft genome sequence of *K. pneumoniae* CICC10011 was annotated with the NCBI Prokaryotic Genomes Automatic Annotation Pipeline (PGAAP). rRNAs were predicted by RNAmmer (12), and tRNAs were predicted by tRNAscan (13).

The draft genome sequence of *K. pneumoniae* CICC10011 comprises 4,883,939 bp, which was assembled into 58 contigs. The *N*_50_ quality measurement of the contigs was 162,338 bp, with a G+C content of 58.4%, and the largest contig assembled was 856,167 bp. The genome sequence was annotated using the PGAAP. The draft genome sequence of CICC10011 contains 5,603 genes, including 3 rRNA genes (5S rRNA, 16S rRNA, and 23S rRNA), and 39 tRNA genes. The annotation results showed that 4,234 proteins have clear biological functions; of these, 3,173 proteins have KEGG orthologs, and 4,433 proteins have COG classifications.

The genome sequence of this promising strain provides significant opportunities to further investigate the metabolic and regulatory mechanisms underlying the formation of 2,3-BD, to explain the genetic reasons for its high productivity and biomass, to analyze the byproducts that may hinder the accumulation of 2,3-BD, and to thoroughly understand the genetic, biological, and physiological characteristics of widely used *K. pneumoniae* strains.

### Nucleotide sequence accession numbers.

This whole-genome shotgun project has been deposited at DDBJ/EMBL/GenBank under the accession no. LBCM00000000. The version described in this paper is the first version, LBCM01000000.

## References

[1] Celińska E, Grajek W 2009 Biotechnological production of 2,3-butanediol—current state and prospects. Biotechnol Adv 27:715–725. doi:10.1016/j.biotechadv.2009.05.002. 19442714

[2] Ji X-J, Huang H, Ouyang P-K 2011 Microbial 2,3-butanediol production: a state-of-the-art review. Biotechnol Adv 29:351–364. doi:10.1016/j.biotechadv.2011.01.007. 21272631

[3] Zeng A-P, Sabra W 2011 Microbial production of diols as platform chemicals: recent progresses. Curr Opin Biotechnol 22:749–757. doi:10.1016/j.copbio.2011.05.005. 21646010

[4] Kim D-K, Rathnasingh C, Song H, Lee HJ, Seung D, Chang YK 2013 Metabolic engineering of a novel *Klebsiella oxytoca* strain for enhanced 2,3-butanediol production. J Biosci Bioeng 116:186–192. doi:10.1016/j.jbiosc.2013.02.021. 23643345

[5] Xu Y, Chu H, Gao C, Tao F, Zhou Z, Li K, Li L, Ma C, Xu P 2014 Systematic metabolic engineering of *Escherichia coli* for high-yield production of fuel bio-chemical 2,3-butanediol. Metab Eng 23:22–33. doi:10.1016/j.ymben.2014.02.004. 24525331

[6] Lee HK, Maddox IS 1986 Continuous production of 2,3-butanediol from whey permeate using *Klebsiella pneumoniae* immobilized in calcium alginate. Enzyme Microb Technol 8:409–411. doi:10.1016/0141-0229(86)90147-X.

[7] Garg SK, Jain A 1995 Fermentative production of 2,3-butanediol: a review. Bioresour Technol 51:103–109. doi:10.1016/0960-8524(94)00136-O.

[8] Petrov K, Petrova P 2009 High production of 2,3-butanediol from glycerol by *Klebsiella pneumoniae* G31. Appl Microbiol Biotechnol 84:659–665. doi:10.1007/s00253-009-2004-x. 19396438

[9] Qin J, Xiao Z, Ma C, Xie N, Liu P, Xu P 2006 Production of 2,3-butanediol by *Klebsiella pneumoniae* using glucose and ammonium phosphate. Chin J Chem Eng 14:132–136. doi:10.1016/S1004-9541(06)60050-5.

[10] Sun L-H, Wang X-D, Dai J-Y, Xiu Z-L 2009 Microbial production of 2,3-butanediol from Jerusalem artichoke tubers by *Klebsiella pneumoniae*. Appl Microbiol Biotechnol 82:847–852. doi:10.1007/s00253-008-1823-5. 19122999

[11] Zerbino DR, Birney E 2008 Velvet: algorithms for *de novo* short read assembly using de Bruijn graphs. Genome Res 18:821–829. doi:10.1101/gr.074492.107. 18349386 PMC2336801

[12] Lagesen K, Hallin P, Rødland EA, Staerfeldt H-H, Rognes T, Ussery DW 2007 RNAmmer: consistent and rapid annotation of ribosomal RNA genes. Nucleic Acids Res 35:3100–3108. doi:10.1093/nar/gkm160. 17452365 PMC1888812

[13] Lowe TM, Eddy SR 1997 tRNAscan-SE: a program for improved detection of transfer RNA genes in genomic sequence. Nucleic Acids Res 25:955–964. doi:10.1093/nar/25.5.0955. 9023104 PMC146525

